# Enhanced Electroluminescence Based on a *π*-Conjugated Heptazine Derivative by Exploiting Thermally Activated Delayed Fluorescence

**DOI:** 10.3389/fchem.2021.693813

**Published:** 2021-05-13

**Authors:** Jie Li, Heqi Gong, Jincheng Zhang, Shiyi Zhou, Li Tao, Lihua Jiang, Qiang Guo

**Affiliations:** ^1^ College of Optoelectronic Technology, Chengdu University of Information Technology, Chengdu, China; ^2^ College of Electrical Engineering & New Energy, Hubei Provincial Engineering Technology Research Center for Power Transmission Line, China Three Gorges University, Yichang, China

**Keywords:** electroluminescence, heptazine, thermally activated delayed fluorescence, organic light-emitting diode, exciplex

## Abstract

Heptazine derivatives have attracted much attention over the past decade by virtue of intriguing optical, photocatalytic as well as electronic properties in the fields of hydrogen evolution, organic optoelectronic technologies and so forth. Here, we report a simple *π*-conjugated heptazine derivative (HAP-3DF) possessing an n→π* transition character which exhibits enhanced electroluminescence by exploiting thermally activated delayed fluorescence (TADF). Green-emitting HAP-3DF shows relatively low photoluminescence quantum efficiencies (*Φ*_p_) of 0.08 in toluene and 0.16 in doped film with bis(2-(diphenylphosphino)phenyl) ether oxide (DPEPO) as the matrix. Interestingly, the organic light-emitting diode (OLED) incorporating 8 wt% HAP-3DF:DPEPO as an emitting layer achieved a high external quantum efficiency (EQE) of 3.0% in view of the fairly low *Φ*_p_ of 0.16, indicating the presence of TADF stemming from n→π* transitions. As the matrix changing from DPEPO to 1,3-di (9*H*-carbazol-9-yl)benzene (mCP), a much higher *Φ*_p_ of 0.56 was found in doped film accompanying yellow emission. More importantly, enhanced electroluminescence was observed from the OLED containing 8 wt% HAP-3DF:mCP as an emitting layer, and a rather high EQE of 10.8% along with a low roll-off was realized, which should be ascribed to the TADF process deriving from exciplex formation.

## Introduction

Organic light-emitting diodes (OLEDs) have received numerous attentions and experienced rapid development in the fields of display and lighting in view of extremely fascinating advantages such as flexibility, thinness, fast response (Im et al., 2017; Choi et al., 2018; Fukagawa et al., 2018). In contrast with traditional fluorescent and phosphorescent materials, pure organic luminophores based on thermally activated delayed fluorescence (TADF) exhibit great potential and better performance with both high electroluminescence (EL) efficiency and low cost (Uoyama et al., 2012; Zhang et al., 2014; Hirata et al., 2015; Sohn et al., 2020). The key design strategy of TADF molecules is to realize a small energy gap (Δ*E*_ST_) between the lowest excited singlet (S_1_) and triplet (T_1_) states through an effective separation of electron densities of the highest occupied molecular orbital (HOMO) and the lowest unoccupied molecular orbital (LUMO) with respect to electron-donating and electron-accepting moieties, respectively (Lee et al., 2012). Current trends in developing highly efficient TADF emitters are mostly focusing on intramolecular donor-acceptor (D-A) type molecules owing to the accompanying small singlet-triplet splitting during the charge-transfer (CT) transitions (Mehes et al., 2012; Zhang et al., 2012; Tanaka et al., 2013; Kawasumi et al., 2015; Chen et al., 2018; Zeng et al., 2018). Alternatively, small Δ*E*_ST_ can be realized by exciplex formation *via* intermolecular CT between electron donors and acceptors, or more localized n→π* transitions involving the lone-pair electrons of heteroatoms and *π* antibonding molecular orbitals (Goushi et al., 2012; Li et al., 2014; Wu et al., 2019; Jeon and Lee, 2020; Zhao et al., 2020).

**FIGURE 1:**
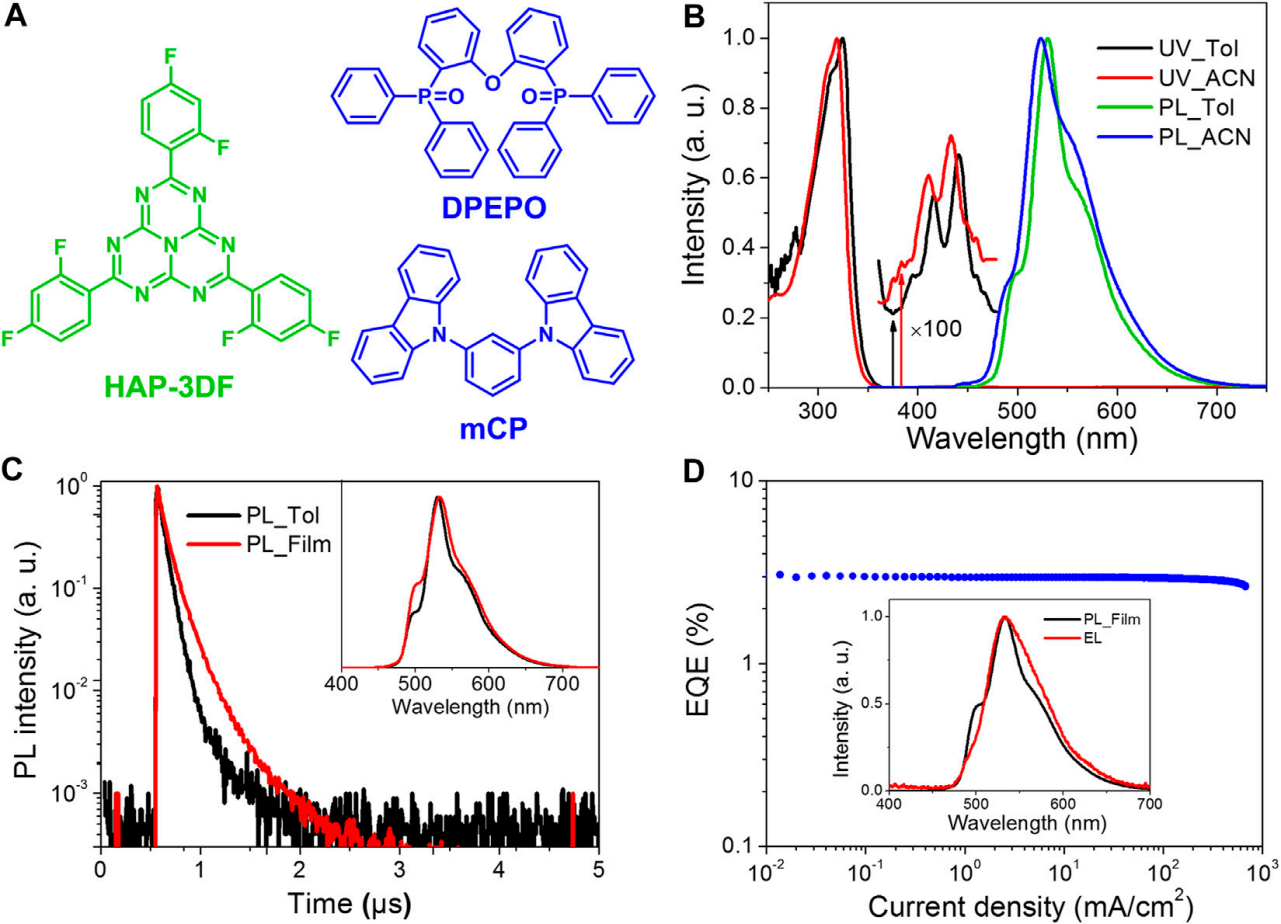
**(A)** Chemical structures of HAP-3DF, DPEPO and mCP. **(B)** UV and PL spectra of HAP-3DF in toluene (Tol) and acetonitrile (ACN). **(C)** Transient PL decay and PL spectra (Inset) of HAP-3DF in toluene (Tol) and DPEPO film (8 wt%). **(D)** EQE characteristics of the OLED incorporating 8 wt% HAP-3DF:DPEPO. Inset: PL spectrum in 8 wt% HAP-3DF:DPEPO and EL spectrum recorded at a current density of 10 mA cm^−2^.

In this work, we report a *π*-conjugated heptazine derivative, 2,5,8-tris(2,4-difluorophenyl)-1,3,4,6,7,9,9b-heptaazaphenalene (HAP-3DF), which exhibits enhanced EL by exploiting n→π* transitions and exciplex-based TADF, respectively. On the basis of the planar and comparatively rigid heterocyclic system with six C=N bonds surrounding a sp^2^-hybridized N atom of the heptazine core, heptazine derivatives have aroused widespread attention over the past decade by virtue of charming optical, photocatalytic as well as electronic properties in the fields of hydrogen evolution, organic optoelectronic technologies and so forth (Ge et al., 2013; Li et al., 2013; Ou et al., 2017; Liu and Ma, 2020). Considering the strong electron-accepting ability of HAP-3DF, bis(2-(diphenylphosphino)phenyl) ether oxide (DPEPO) with two electron-accepting diphenylphosphine oxide groups was chosen as the host material. Encouragingly, the OLED incorporating 8 wt% HAP-3DF:DPEPO as an emitting layer achieved a high maximum external quantum efficiency (EQE) of 3.0% in comparison to the fairly low photoluminescence quantum efficiency (*Φ*_p_) of 0.16, indicating the presence of TADF stemming from n→π* transitions. To realize exciplex-based TADF, 1,3-di (9*H*-carbazol-9-yl)benzene (mCP) with two electron-donating carbazole moieties was chosen as the electron-donating material for HAP-3DF. More importantly, enhanced EL was observed from the OLED containing 8 wt% HAP-3DF:mCP as an emitting layer, and a rather high EQE of 10.8% along with a low roll-off was realized, which should be assigned to the TADF process deriving from exciplex formation. The chemical structures of HAP-3DF, DPEPO and mCP are depicted in Figure 1A.

## Results and Discussion

Details of synthetic routes and quantum chemical calculation of HAP-3DF are described in the Supplementary Material. The HOMO and LUMO of HAP-3DF are depicted in Supplementary Figure S1. The HOMO is mainly distributed over the sp^2^-hybridized N atoms in the heptazine core while the LUMO spreads to the whole *π*-conjugated system, which is in accordance with the character of n→π* transition. Based on the optimized geometry structure, the energies of S_1_ and T_1_ were calculated to be 2.7753 eV (HOMO→LUMO) and 2.6022 eV (HOMO→LUMO), respectively, indicating that both S_1_ and T_1_ possess n→π* transition characteristics. Meanwhile, the Δ*E*_ST_ is calculated to be as small as 0.1731 eV, implying that n→π* transition could be an efficient TADF pathway.

To further confirm the n→π* transition character of HAP-3DF, the ultraviolet–visible absorption (UV) and photoluminescence (PL) spectra were recorded in toluene and a more polar solvent, acetonitrile, respectively (Figure 1B). The intense absorption band centered around 320 nm is assigned to *π*→π* electronic transition with regard to the *π*-conjugated system. It is noteworthy that there is an additional low-energy band centered around 450 nm, which should be attributed to the n→π* transition involving the lone-pair electrons of N heteroatoms and a *π* antibonding molecular orbital. As expected, both UV and PL spectra of HAP-3DF in acetonitrile showed typical blue shifts compared with that in toluene, indicating typical n→π* transition characteristics due to the stabilization effect of polar acetonitrile molecules on lone-pair electrons of N atoms (Sidman, 1958; Goodman, 1961). Moreover, both the long-wavelength absorption and emission bands show well-resolved vibronic structures, which should be ascribed to the rigid molecular structure of HAP-3DF. Transient PL decay of HAP-3DF in oxygen-free toluene in the time range of 5 μs is shown in Figure 1C, and it presented a prompt fluorescence lifetime of 70 ns and a delayed one of 290 ns The radiative rate constant (*k*_r_) of S_1_ can be calculated to be 1.1 × 106 s^−1^ combining with the corresponding *Φ*_p_ of 0.08. The extremely low fluorescence rate and *Φ*_p_ are in good agreement with the typical characteristics of n→π* transitions. Further, transient PL decay and emission spectrum of the 8 wt% HAP-3DF:DPEPO doped film in vacuum were measured (Figure 1C), and a more intense delayed component and a higher *Φ*_p_ of 0.16 were observed, probably because of the suppressed nonradiative decay due to the rigid and tightly packed environment. Meanwhile, the well-resolved PL spectrum centered at 530 nm is quite similar to that in toluene, indicating that all photons are generated from the same excited singlet state of HAP-3DF in the doped film.

To get better insights into EL characteristics of HAP-3DF, an OLED containing 8 wt% HAP-3DF:DPEPO as an emitting layer was fabricated. The device structure was ITO/α-NPD (35 nm)/TCTA (5 nm)/8 wt% HAP-3DF:DPEPO (15 nm)/DPEPO (5 nm)/TPBI (40 nm)/LiF (0.8 nm)/Al (80 nm), where ITO is indium tin oxide, α-NPD is *N*,*N′*-di (naphthalen-1-yl)-*N*,*N′*-diphenylbenzidine as a hole transport layer, TPBI is 1,3,5-tris(*N*-phenylbenzimidazole-2-yl)benzene as an electron transport layer, and LiF and Al act as the cathode. Thin tris(4-(9H-carbazol-9-yl)phenyl)amine (TCTA) and DPEPO layers were inserted to block electrons from the cathode and holes from the anode, respectively, and simultaneously confine the excitons in the emitting layer. The OLED structure and energy diagram are depicted in Supplementary Figure S2. The EL performance are shown in Figure 1D and Supplementary Figures S3, S4. The EL spectra measured at 1, 10 and 100 mA cm^−2^ are well overlapped with a maximum emission peak of 530 nm, and in good accordance with the PL spectrum of the emitting layer. More importantly, without any light out-coupling enhancement architechure, the OLED showed a comparatively high maximum EQE of 3.0% along with a rather low roll-off considering the fairly low *Φ*_p_ of 0.16. The theoretical maximum EQE can be calculated from the following formula, EQE = *γ* × *η*_r_ × PLQY × *η*_out_, where *γ* is the electron/hole recombination ratio, *η*_r_ is the exciton formation ratio for radiative transitions (*η*_r_ = 0.25 for conventional fluorescent emitters), and *η*_out_ is the light out-coupling efficiency. Accordingly, the theoretical maximum EQE should be limited to 0.8–1.2% assuming that HPM-3DF is a conventional fluorescent molecule with *γ* = 1.0, *η*_r_ = 0.25, PLQY = 0.16 and *η*_out_ = 0.2–0.3. Thus, the high EL efficiency should be ascribed to the effective TADF process in HAP-3DF based on n→π* transitions by harvesting radiative singlet excitons upon efficient up-conversion of abundant triplet excitons under electrical excitation.

**FIGURE 2:**
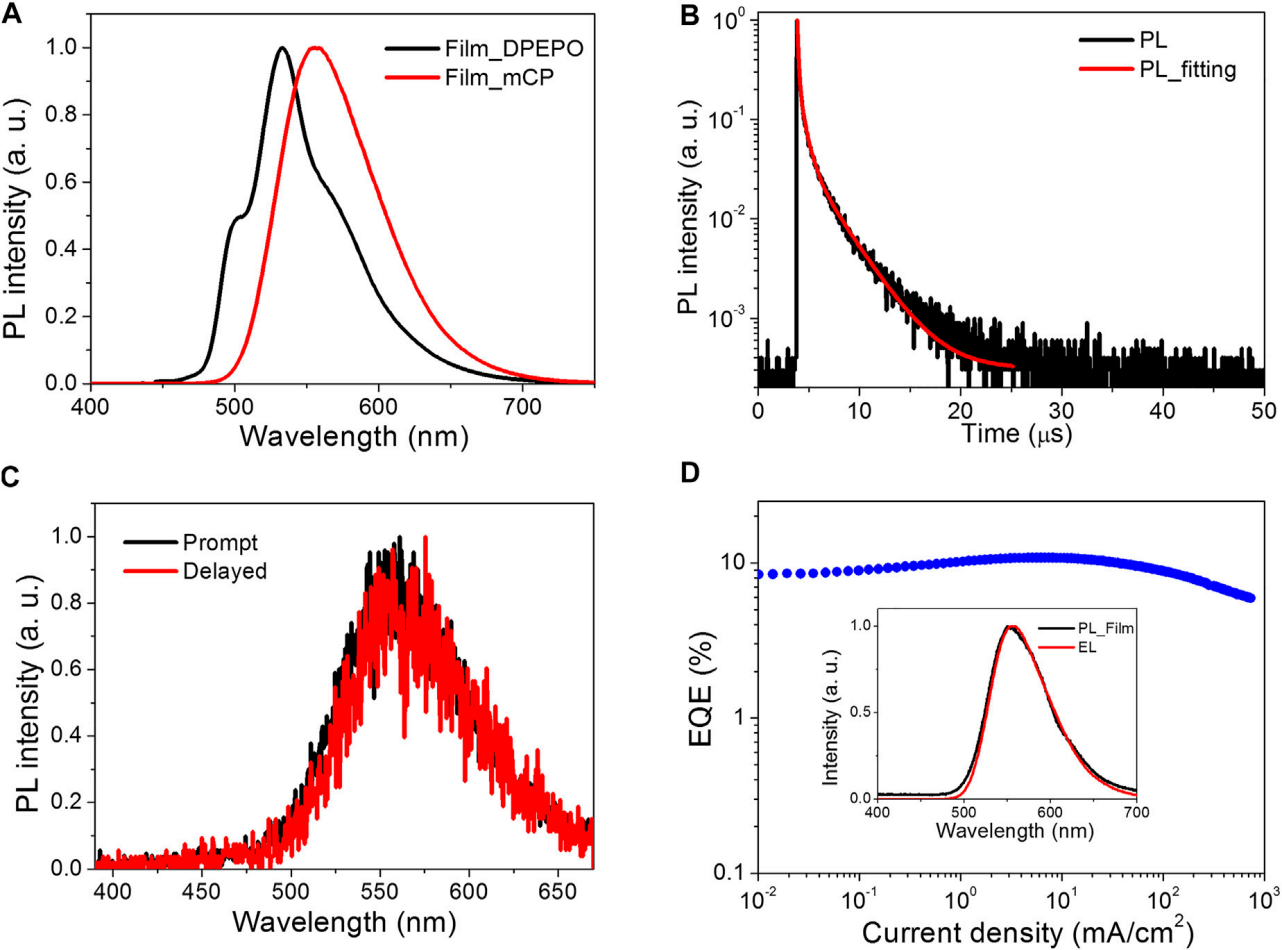
**(A)** PL spectra of 8 wt% HAP-3DF in DPEPO and mCP. **(B)** Transient PL decay of 8 wt% HAP-3DF:mCP. **(C)** Prompt and delayed PL spectra of 8 wt% HAP-3DF:mCP at 300 K. **(D)** EQE as a function of current density. Inset: PL spectrum in 8 wt% HAP-3DF:mCP and EL spectrum recorded at a current density of 10 mA cm^−2^.

On the basis of the strong electron-accepting ability of HAP-3DF, exciplex-based TADF could be anticipated by mixing with materials with the electron donating character. The exciplex is an excited complex produced by *π*→π* interactions between adjacent conjugated linkers or between a linker and a guest molecule, typically exhibiting broad, featureless luminescence (Allendorf et al., 2009). As is well-known, there is usually an obviously red-shifted and broadened emission spectrum for exciplexes compared with corresponding pure emitters (Goushi et al., 2012; Hung et al., 2013). Herein, as a widely used host material, mCP was chosen as the electron donor on account of the two electron-donating carbazole moieties. An optimized exciplex system of 8 wt% HAP-3DF:mCP was fabricated and characterized. The PL spectra and transient PL decay of 8 wt% HAP-3DF:mCP are presented in Figure 2A, respectively. In contrast, the PL spectrum of 8 wt% HAP-3DF:mCP exhibits typical exciplex characteristics with a broad and featureless band centered at 556 nm, which is 26 nm red-shifted compared with that of 8 wt% HAP-3DF:DPEPO. Meanwhile, the full width at half maximum (FWHM) of the exciplex system is 79 nm, which is only 12 nm larger than that of HAP-3DF alone (67 nm), and the narrow FWHM is beneficial to the color purity of OLEDs. Excitingly, the exciplex system shows a remarkably high *Φ*_p_ of 0.56, which is much higher than that of 8 wt% HAP-3DF:DPEPO. The narrow FWHM and high *Φ*_p_ should be associated with the rigid geometries and tight molecular packing of HAP-3DF and mCP, which can effectively confine molecular motions, suppress the nonradiative transition of singlet and triplet exciplex excitons, and endow the blend film with high PL performance. Figure 2B shows the transient PL decay of an 8 wt% HAP-3DF:DPEPO film in vacuum condition at 300 K. Obviously, the transient decay process can be divided into prompt and delayed components. The prompt component with the lifetime of 92 ns should be assigned to conventional fluorescence-based exciplex emission, while the two delayed components with lifetimes of 617 ns and 2.75 μs are generated from the exciplex-based TADF involving an up-conversion process of triplet excitons from T_1_ to S_1_ (Goushi et al., 2012). To better elucidate the exciplex mechanism, prompt and delayed PL spectra of 8 wt% HAP-3DF:mCP at 300 K were characterized (Figure 2C). The well-overlapped prompt and delayed emission spectra confirm that all photons stemmed from the same excited state. Moreover, 6-dicarbazolo-1,5-pyridine (PYD2) was chosen as another electron-donating material in view of the similar molecular structure with mCP (Supplementary Figure S5). The photophysical characteristics of 8 wt% HAP-3DF:PYD2 film at 300 K were measured and shown in Supplementary Figure S5 and Table S2. In comparison to the 8 wt% HAP-3DF:mCP exciplex system, the 8 wt% HAP-3DF:PYD2 film shows quite similar PL spectrum, indicating the formation of exciplex between HAP-3DF and PYD2 molecules. Meanwhile, the obvious delayed emission and well-overlapped prompt and delayed components further verify the presence of exciplex formation. Notably, the 8 wt% HAP-3DF:PYD2 exciplex film exhibits a lower *Φ*_p_ of 0.39, which might be associated with the subtly changing electron-donating ability of HAP-3DF due to the introduction of pyridine, and the variance of intermolecular interactions between HAP-3DF and PYD2 in comparison to that of HAP-3DF and mCP.

To verify the EL performance of the exciplex system, an OLED based on the 8 wt% HAP-3DF:mCP exciplex system was fabricated with a structure of ITO/α-NPD (35 nm)/TCTA (5 nm)/8 wt% HAP-3DF:mCP (15 nm)/DPEPO (5 nm)/TPBI (40 nm)/LiF (0.8 nm)/Al (80 nm). The OLED structure and energy diagram are depicted in Supplementary Figure S6. The EL performance are shown in Figure 2D and Supplementary Figures S7, S8. The EL spectra measured at 1, 10 and 100 mA cm^−2^ are well overlapped and almost identical to the PL spectrum of an 8 wt% HAP-3DF:mCP blend film. The photon energy of the exciplex was calculated to be 2.5 eV from the onset of the EL spectrum (492 nm), which is consistent with the energy difference between the LUMO of HAP-3DF (−3.4 eV) and HOMO of mCP (−5.9 eV) (Supplementary Figure S9). From the current density–voltage–luminance (*J*–*V*–*L*) characteristics, the OLED exhibited a considerably high luminance of 18,478 cd m^−2^ at 12.4 V. Encouragingly, a remarkably high EQE of 10.8% was realized along with a rather low roll-off (10.8% at 1000 cd m^−2^, 9.6% at 5000 cd m^−2^, 8.5% at 10,000 cd m^−2^), which significantly exceeds the theoretical maximum EQE (2.8–4.2%) if the 8 wt% HAP-3DF:mCP system is a conventional fluorescent emitter. Overall, the excellent EL performance is partially attributed to the well-balanced electron and hole fluxes into the emitting zone. Meanwhile, the rigid geometries and tight molecular packing of HAP-3DF and mCP molecules are beneficial to effectively confining molecular motions and suppressing the nonradiative transition of singlet and triplet excitons. More importantly, it should be ascribed to the efficient up-conversion of triplet exciplex excitons from T_1_ to S_1_ through TADF process which endows the OLED with high EL performance.

## Conclusion

In summary, we report a *π*-conjugated heptazine derivative (HAP-3DF) exhibiting enhanced EL by exploiting n→π* transition and exciplex-based TADF, respectively. To realize TADF through two different processes, DPEPO with two electron-accepting diphenylphosphine oxide groups and mCP with two electron-donating carbazole moieties, were chosen as the host materials for HAP-3DF. Encouragingly, the OLED incorporating 8 wt% HAP-3DF:DPEPO as an emitting layer achieved a high EQE of 3.0% in comparison to the fairly low *Φ*_p_ of 0.16, indicating the presence of efficient TADF stemming from n→π* transitions. More importantly, enhanced EL was observed from the OLED containing 8 wt% HAP-3DF:mCP as an emitting layer, and a remarkably high EQE of 10.8% along with a fairly low roll-off (10.8% at 1000 cd m^−2^, 9.6% at 5000 cd m^−2^, 8.5% at 10,000 cd m^−2^) was realized, which should be assigned to the TADF process deriving from exciplex formation. These findings are of fundamental interest for the development of highly efficient OLEDs based on n→π* transitions and exciplex systems.

## Data Availability

The original contributions presented in the study are included in the article/Supplementary Material, further inquiries can be directed to the corresponding author.
