# Supplementary material for: Enhanced Electroluminescence Based on a π-Conjugated Heptazine Derivative by Exploiting Thermally Activated Delayed Fluorescence
Source: Front Chem. 2021 May 13;9:693813. doi: 10.3389/fchem.2021.693813 (PMC8155250; doi:10.3389/fchem.2021.693813)
Supplement: Supplementary file 1 [file DataSheet1.pdf]

Supplementary Material

Enhanced Electroluminescence Based on a π-Conjugated Heptazine Derivative by Exploiting Thermally Activated Delayed Fluorescence

Jie Li[1], Heqi Gong[1], Jincheng Zhang[1], Shiyi Zhou[1], Li Tao[1], Lihua Jiang[2] and Qiang Guo[1*]

[1]College of Optoelectronic Technology, Chengdu University of Information Technology, Chengdu, China

[2] College of Electrical Engineering & New Energy, Hubei Provincial Engineering Technology Research Center for Power Transmission Line, China Three Gorges University, Yichang, China

*** Correspondence:** qiangguo@cuit.edu.cn (Qiang Guo)

Table of Contents

1 General Methods

2 Synthetic Routes of HAP-3DF

3 Quantum Chemical Calculations

4 Calculation of the radiative rate constant (k_r) of HAP-3DF

5 PL and EL Performance

6 ^{1}H NMR Spectrum of HAP-3DF

1 General Methods

The UV and PL spectra were recorded with a Shimadzu UV-2550 spectrophotometer and Shimadzu RF-5301PC fluorescence spectrometer, respectively. The Φ_p and transient PL decay were recorded using a Hamamatsu C9920-02 and a Hamamatsu C4334 measurement system, respectively. The ^{1}H NMR spectrum was obtained with a Bruker AVANCE III 400 NMR spectrometer. HAP-3DF was not sufficiently soluble in common deuterated solvents, so its ^{13}C NMR spectrum was not obtained. High resolution mass spectrometry (HRMS) by fast atom bombardment was conducted using a JEOL JMS-700 spectrometer. Elemental analysis was performed with a Yanaco MT-5 elemental analyzer. The OLED was fabricated by vacuum thermal evaporation under a pressure lower than 4×10^{-4} Pa. OLEDs were prepared on a glass substrate pre-coated with a 150 nm-thick ITO layer. Prior to the deposition of the organic layers, the substrate was cleaned with ultra-purified water and organic solvents, treated with UV-ozone for 15 min, and finally transferred to a vacuum deposition system for organic and metal deposition. The intersection of ITO and the metal electrodes gave an active device area of 4 mm^2. The OLED device was characterized under atmospheric conditions without any encapsulation or light out-coupling enhancement. The current density-voltage-luminance (*J-V-L*) characteristics of the OLED were measured with an Agilent E5273A semiconductor parameter analyzer and a Newport 1930C optical power meter. EL spectra were recorded using an Ocean Optics USB2000multi-channel spectrometer. All spectrophotometric grade solvents and starting materials were purchased and used as received unless otherwise noted. All reactions were carried out under N_2 atmosphere.

2 Synthetic Routes of HAP-3DF

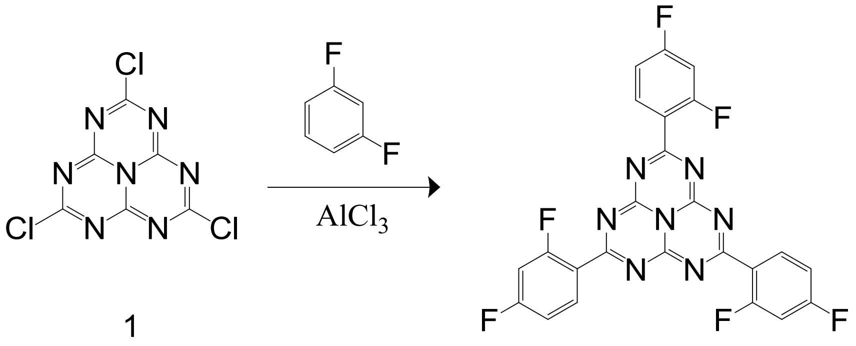

2,5,8-trichloro-1,3,3a[1],4,6,7,9-heptaazaphenalene (1): The synthesis of compound 1 was performed according to the synthetic method reported previously [S1].

2,5,8-tris(2,4-difluorophenyl)-1,3,4,6,7,9,9b-heptaazaphenalene (HAP-3DF): Compound 1 (1.0 g, 3.6 mmol) was added to a solution of $AlCl_3$ (2.41 g, 18 mmol) in 1,3-difluorobenzene (10 ml) at 0 °C. The mixture was stirred at 0 °C for 30 min and then at 80 °C for 8 h. The reaction mixture was cooled to room temperature and then ice was added and stirred for 1 h. Subsequently, the mixture was heated at 100 °C for 1 h and then cooled to room temperature, filtered, washed with H_2O, taken up in toluene and pre-adsorbed onto silica. These pre-adsorbed materials were subjected to flash chromatography (EtOAc/toluene, 0:100 v/v and then 10:90 v/v) to give HAP-3DF (0.46 g, yield: 25%) as a yellow-green solid. This compound was further purified by sublimation. ^{1}H NMR (400 MHz, $CDCl_3$): δ 8.36-

8.42 (m, 3H), 6.92-7.00 (m, 3H), 1.59 (s, 3H). HRMS (m/z):[M]$^-$ calcd for $C_{24}H_9F_6N_7$ 509.0824, found 509.0820. Elemental anal. calcd for $C_{24}H_9F_6N_7$ (%): C 56.59, H 1.78, N 19.25; found: C 56.50, H 1.73, N 19.33.

3 Quantum Chemical Calculations

All calculations were performed using the Gaussian 09 program package. The HOMO and LUMO of HAP-3DF were calculated using the nonlocal density functional of Becke's 3-parameters employing Lee-Yang-Parr functional (B3LYP) with 6-31G(d) basis sets. The S_1 and T_1 were calculated by the TD-DFT method at the optimized ground-state geometries using the B3LYP mode with a 6-31G (d) basis set [S2].

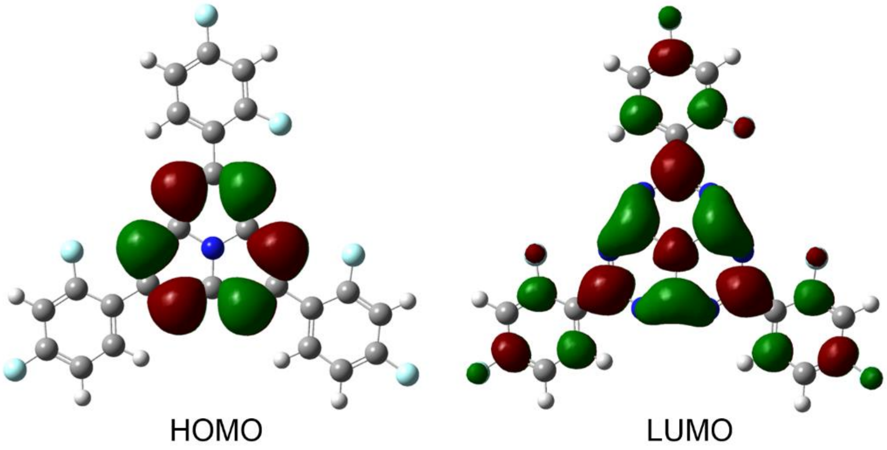

Figure S1. The HOMO and LUMO of HAP-3DF calculated at the B3LYP/6-31(d) level.

3.1 Optimized Geometry Data for HAP-3DF (unit: Å)

C	0.915558	3.941033	-0.000337
C	0.593434	2.499930	-0.000228
N	1.604350	1.618745	-0.000334
C	1.293802	0.323474	-0.000209
N	-0.056736	-0.059539	-0.000008
C	-1.064942	0.917294	-0.000036
N	-0.720514	2.199754	-0.000118
N	2.238866	-0.614638	-0.000298
C	1.842154	-1.896075	-0.000032
N	0.567817	-2.332268	0.000190
C	-0.400295	-1.420734	0.000125
N	-1.684500	-1.764801	0.000218
C	-2.600331	-0.776489	0.000151
N	-2.343529	0.539215	0.000017
C	-4.010920	-1.216969	0.000126
C	2.876398	-2.950785	-0.000089
C	2.225985	4.463133	0.000558
C	2.477042	5.831123	0.000347
C	1.396006	6.700952	-0.000776

C	0.079450	6.245794	-0.001653
C	-0.141244	4.876773	-0.001399
C	-5.117506	-0.342717	0.001212
C	-6.428307	-0.807800	0.001064
C	-6.642998	-2.178594	-0.000200
C	-5.591351	-3.091942	-0.001269
C	-4.294977	-2.599300	-0.001062
C	4.265059	-2.702564	0.000937
C	5.200378	-3.732135	0.000772
C	4.741681	-5.041494	-0.000441
C	3.383151	-5.349767	-0.001443
C	2.473320	-4.303253	-0.001226
F	1.632870	8.022393	-0.001000
F	3.296805	3.665717	0.001771
F	-7.906409	-2.632763	-0.000373
F	-4.961998	0.984021	0.002563
F	5.640349	-6.038882	-0.000629
F	4.754422	-1.460527	0.002249
H	3.496370	6.197970	0.001077
H	-0.738939	6.956899	-0.002503
H	-1.151634	4.486134	-0.002029
H	-7.254936	-0.107557	0.001949
H	-5.798900	-4.156073	-0.002233
H	-3.452080	-3.279620	-0.001832
H	6.259756	-3.505514	0.001609
H	3.063423	-6.385731	-0.002363
H	1.409018	-4.504746	-0.001942

3.2 Excitation Energies and Oscillator Strengths for HAP-3DF

```
Excited State   1:      Triplet-A      2.6022 eV  476.46 nm  f=0.0000  <S**2>=2.000
     128 ->129         0.70219
 Excited State   2:      Singlet-A      2.7753 eV  446.75 nm  f=0.0001  <S**2>=0.000
     128 ->129         0.70211
 Excited State   3:      Triplet-A      3.0436 eV  407.35 nm  f=0.0000  <S**2>=2.000
     125 ->131         0.15865
     126 ->131        -0.10223
     127 ->129         0.59311
     127 ->131         0.16454
 Excited State   4:      Triplet-A      3.0641 eV  404.63 nm  f=0.0000  <S**2>=2.000
     125 ->129        -0.12194
     125 ->130         0.17581
     126 ->129         0.57270
     126 ->131        -0.12493
     127 ->130        -0.11027
     127 ->131        -0.14850
 Excited State   5:      Triplet-A      3.1046 eV  399.36 nm  f=0.0000  <S**2>=2.000
```

```
   121 ->129       -0.17628
   125 ->129        0.49889
   126 ->129        0.14063
   126 ->130        0.29727
   126 ->135       -0.10596
   127 ->131        0.17661
Excited State   6:      Triplet-A      3.1466 eV  394.03 nm  f=0.0000  <S**2>=2.000
   128 ->130        0.68742
   128 ->135        0.10815
Excited State   7:      Triplet-A      3.2318 eV  383.64 nm  f=0.0000  <S**2>=2.000
   128 ->131        0.68509
   128 ->136        0.10798
Excited State   8:      Singlet-A      3.3157 eV  373.93 nm  f=0.0000  <S**2>=0.000
   119 ->130        0.10980
   124 ->129        0.68904
Excited State   9:      Singlet-A      3.3668 eV  368.25 nm  f=0.0000  <S**2>=0.000
   120 ->129        0.68756
Excited State  10:      Singlet-A      3.4186 eV  362.68 nm  f=0.0000  <S**2>=0.000
   119 ->129        0.67514
   124 ->130        0.17269
Excited State  11:      Singlet-A      3.4756 eV  356.72 nm  f=0.3943  <S**2>=0.000
   127 ->129        0.70092
Excited State  12:      Singlet-A      3.5613 eV  348.14 nm  f=0.4334  <S**2>=0.000
   126 ->129        0.70238
```

4 Calculation of the radiative rate constant (k_r) of HAP-3DF

Based on the transient PL decay parameters and the PL quantum efficiency (Φ_p) in **Table S1**, the PL quantum efficiency of the prompt component (Φ_{prompt}) and the radiative rate constant of the singlet excited state (k_r) can be calculated by following equations:

$$\Phi_{prompt} = \frac{\tau_1 A_1}{\tau_1 A_1 + \tau_2 A_2} \Phi_p \quad \text{(S1)}$$

$$k_r = \frac{\Phi_{prompt}}{\tau_1} \quad \text{(S2)}$$

Table S1 The details of transient PL decay parameters and Φ_p of HAP-3DF in oxygen-free toluene.

Compound	τ_1 (ns)	A_1	τ_2 (ns)	A_2	Φ_p
HAP-3DF	70	37740	290	474	0.08

5 PL and EL Performance

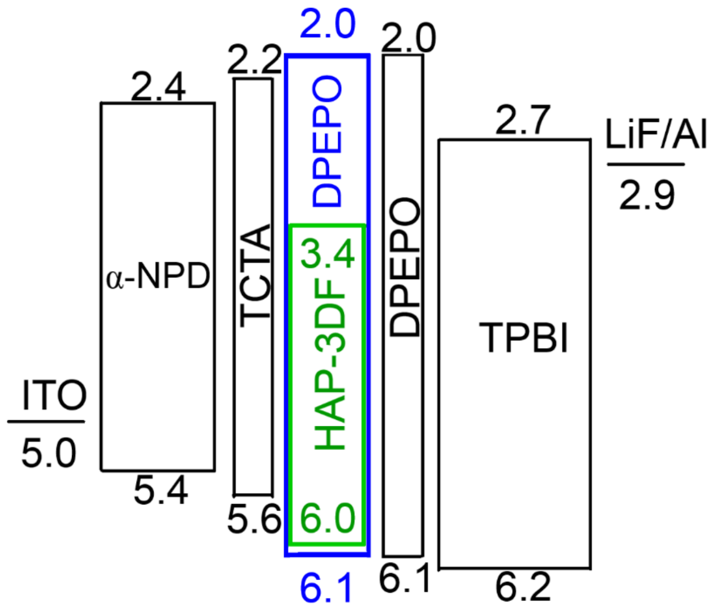

Figure S2. Energy diagram of the OLED incorporating 8 wt% HAP-3DF:DPEPO as an emitting layer.

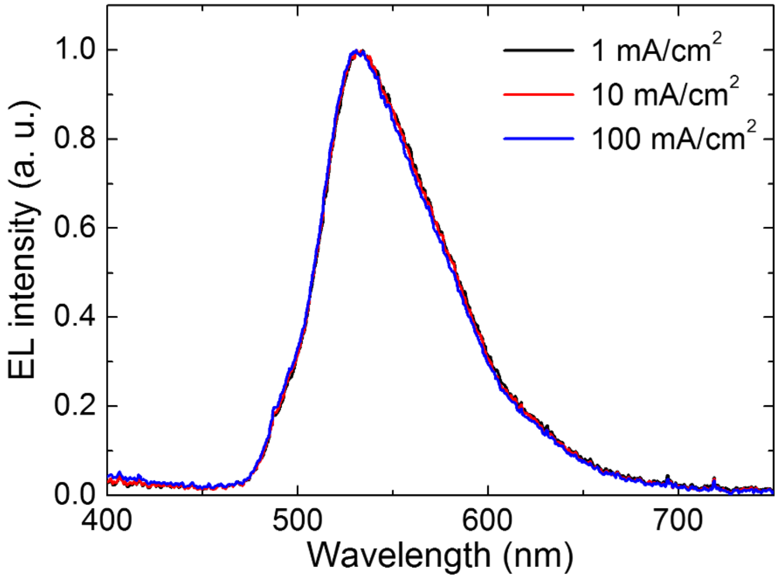

Figure S3. EL spectra of the OLED containing 8 wt% HAP-3DF:DPEPO as an emitting layer recorded at various densities.

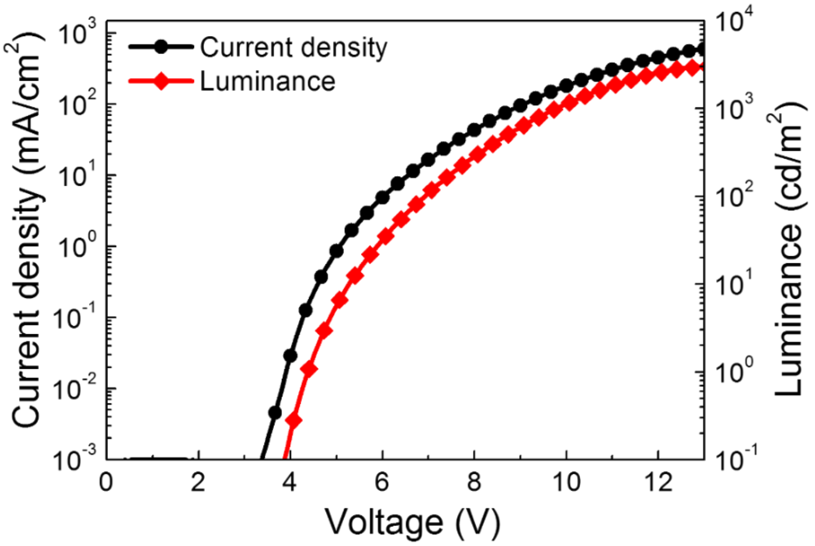

Figure S4. *J*-*V*-*L* characteristics of the OLED containing 8 wt% HAP-3DF:DPEPO as an emitting layer.

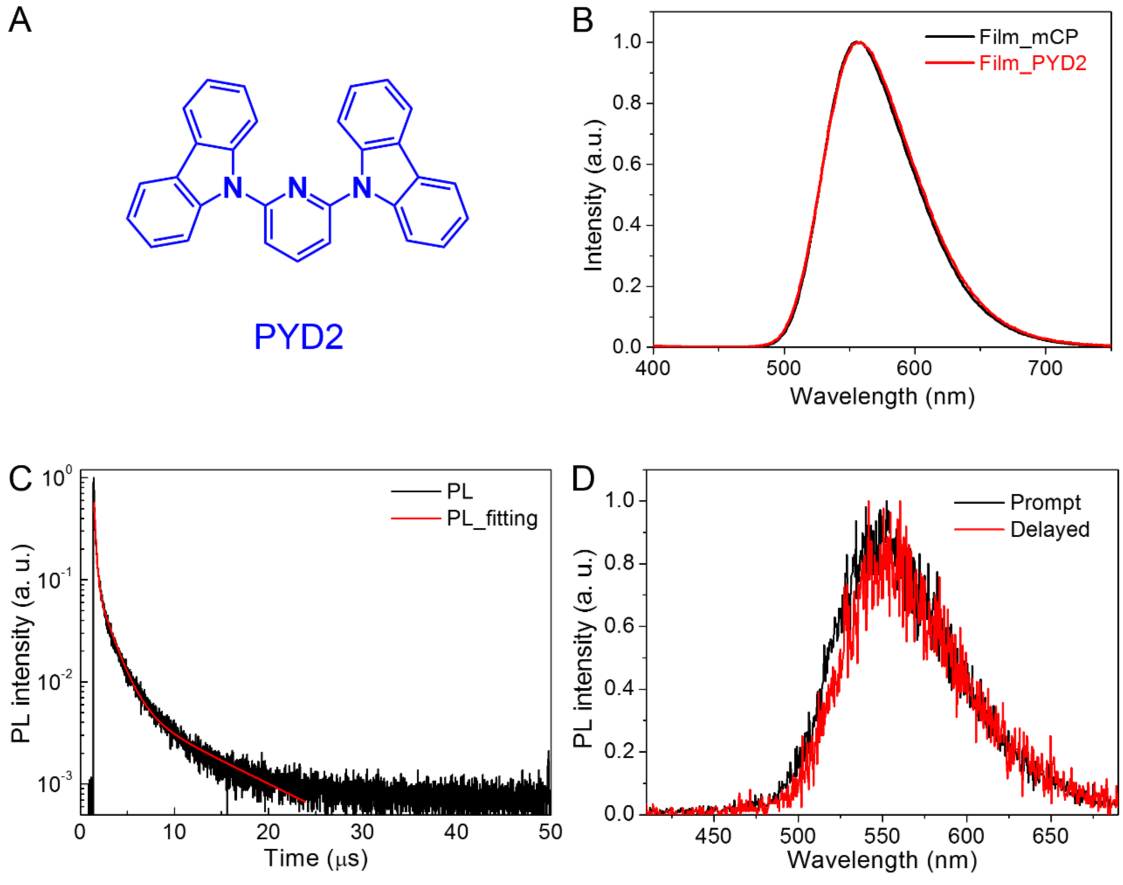

Figure S5. (A) Chemical structures of PYD2. **(B)** PL spectra of 8 wt% HAP-3DF:mCP and 8 wt% HAP-3DF:PYD2 films. **(C)** Transient PL decay of 8 wt% HAP-3DF:PYD2 film in vacuum at 300 K. **(D)** Prompt and delayed PL spectra of 8 wt% HAP-3DF:PYD2 at 300 K.

Table S2 The details of photophysical characteristics of 8wt% HAP-3DF:PYD2 in vacuum at 300K.

Compound	λ_{em} (nm)[a]	τ_1 (ns)	τ_2 (μs)	τ_3 (μs)	Φ_p
HAP-3DF	558	185	1.44	9.58	0.39

[a] The wavelength of emission peak.

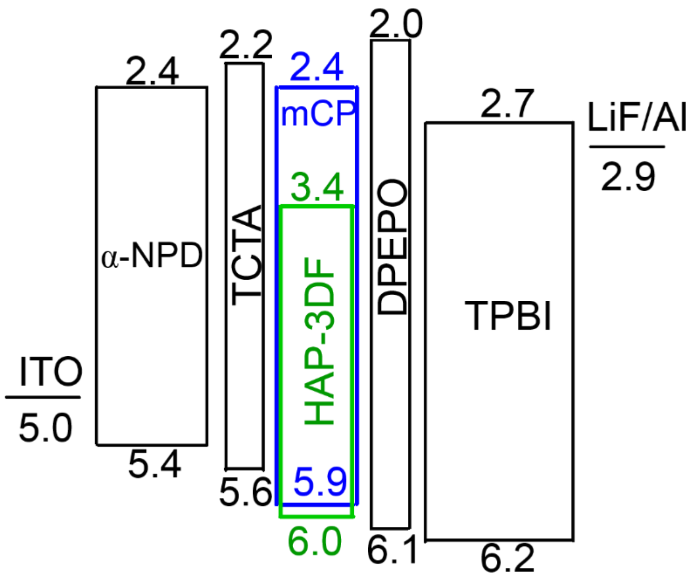

Figure S6. Energy diagram of the OLED incorporating 8 wt% HAP-3DF:mCP as an emitting layer.

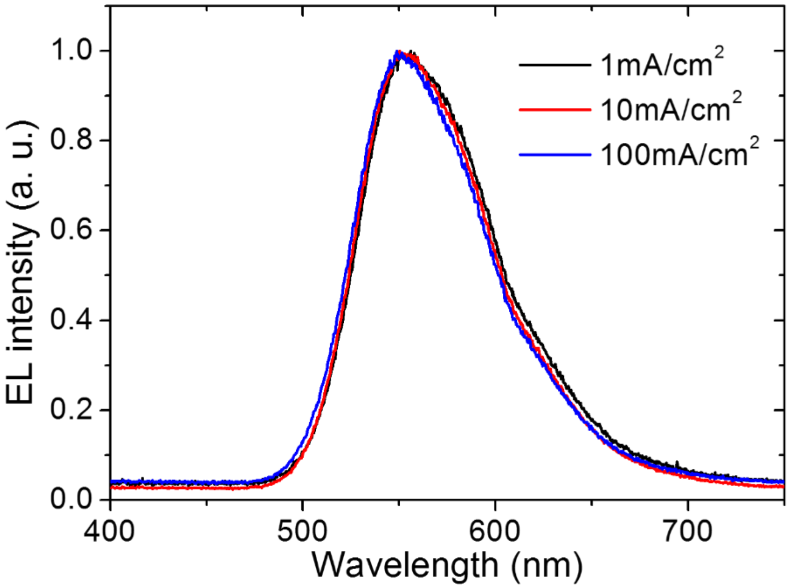

Figure S7. EL spectra of the OLED containing 8 wt% HAP-3DF:mCP as an emitting layer recorded at various densities.

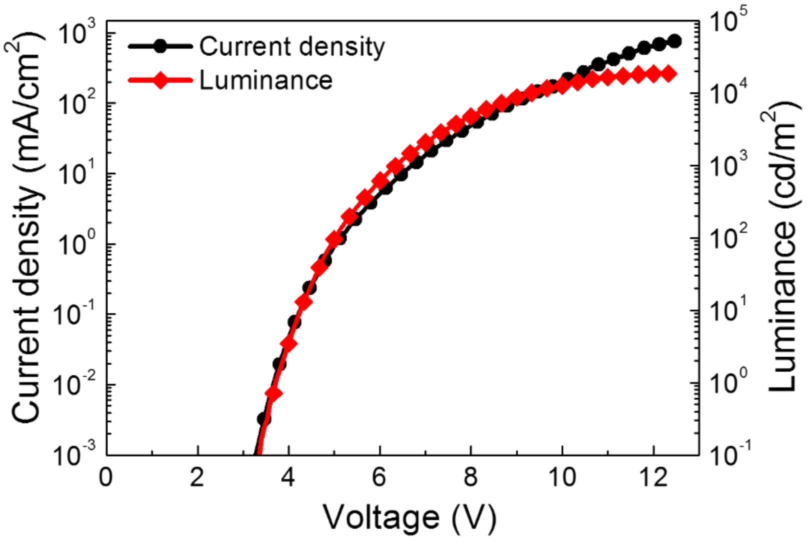

Figure S8. *J*-*V*-*L* characteristics of the OLED containing 8 wt% HAP-3DF:mCP as an emitting layer.

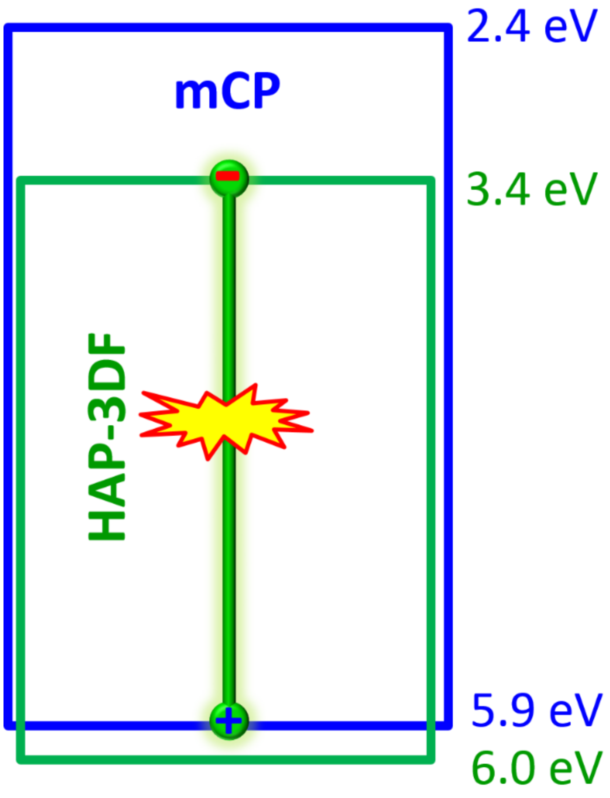

Figure S9. The energy diagram of the 8 wt% HAP-3DF:mCP exciplex system.

6 ^{1}H NMR Spectrum of HAP-3DF

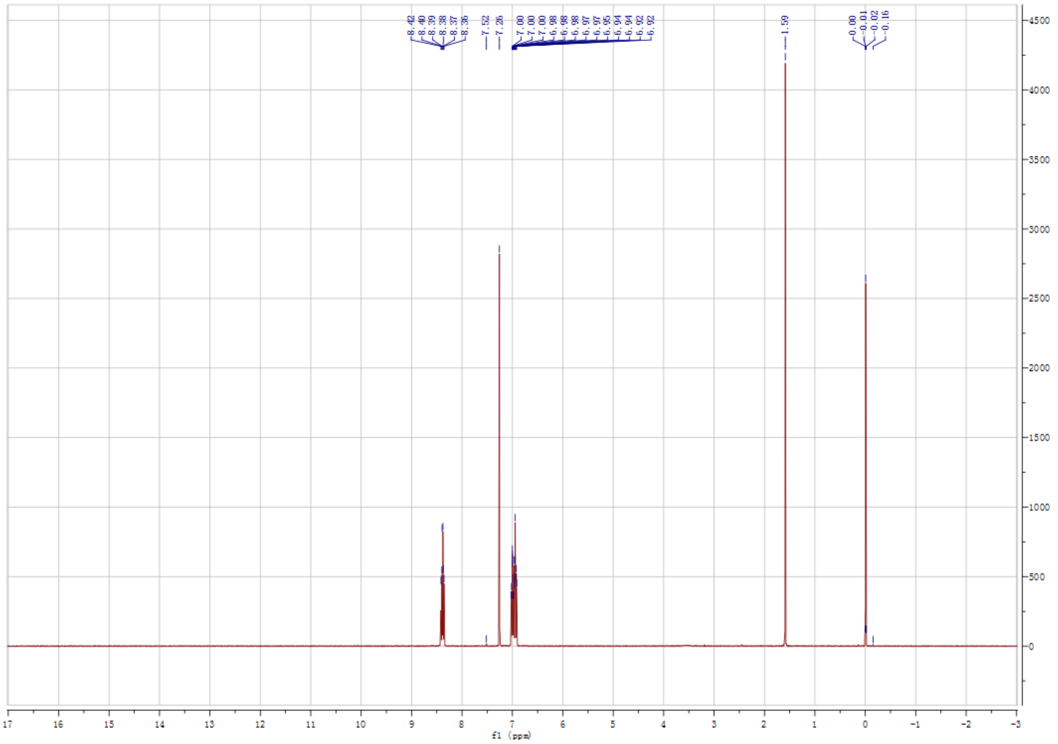

Figure S10. ^{1}H NMR spectrum of HAP-3DF in $CDCl_3$.

REFERENCES

S1 J. Li, T. Nakagawa, J. MacDonald, Q. Zhang, H. Nomura, H. Miyazaki and C. Adachi, Adv. Mater. 25, 3319 (2013).

S2 M. J. Frisch, G. W. Trucks, H. B. Schlegel, G. E. Scuseria, M. A. Robb, J. R. Cheeseman, G. Scalmani, V. Barone, B. Mennucci, G. A. Petersson, H. Nakatsuji, M. Caricato, X. Li, H. P. Hratchian, A. F. Izmaylov, J. Bloino, G. Zheng, J. L. Sonnenberg, M. Hada, M. Ehara, K. Toyota, R. Fukuda, J. Hasegawa, M. Ishida, T. Nakajima, Y. Honda, O. Kitao, H. Nakai, T. Vreven, J. A. Montgomery, Jr., J. E. Peralta, F. Ogliaro, M. Bearpark, J. J. Heyd, E. Brothers, K. N. Kudin, V. N. Staroverov, R. Kobayashi, J. Normand, K. Raghavachari, A. Rendell, J. C. Burant, S. S. Iyengar, J. Tomasi, M. Cossi, N. Rega, J. M. Millam, M. Klene, J. E. Knox, J. B. Cross, V. Bakken, C. Adamo, J. Jaramillo, R. Gomperts, R. E. Stratmann, O. Yazyev, A. J. Austin, R. Cammi, C. Pomelli, J. W. Ochterski, R. L. Martin, K. Morokuma, V. G. Zakrzewski, G. A. Voth, P. Salvador, J. J. Dannenberg, S. Dapprich, A. D. Daniels, Ö. Farkas, J. B. Foresman, J. V. Ortiz, J. Cioslowski, and D. J. Fox, *Gaussian 09, Revision C.01*. (Gaussian, Inc., 2009).
